# Vaccine Effectiveness against SARS-CoV-2 Variant P.1 in Nursing-Facility Residents, Washington, USA, April 2021

**DOI:** 10.3201/eid2811.221043

**Published:** 2022-11

**Authors:** James W. Lewis, Julie Loughran, Li Deng, Jasmine Varghese, Shauna Clark, Casandra Harrison, Molly Gacetta, John A. Jernigan, Katherine E. Fleming-Dutra

**Affiliations:** Public Health Seattle and King County, Seattle, Washington, USA (J.W. Lewis, J. Loughran, S. Clark, C. Harrison, M. Gacetta);; Centers for Disease Control and Prevention, Atlanta, Georgia, USA (L. Deng, J. Varghese, J.A. Jernigan. K.E. Fleming-Dutra)

**Keywords:** COVID-19, SARS-CoV-2, P.1, vaccine effectiveness, long-term care, respiratory infections, zoonoses, viruses, United States, coronavirus disease, severe acute respiratory syndrome coronavirus 2

## Abstract

A SARS-CoV-2 P.1 (Gamma) variant outbreak occurred at a skilled nursing facility in Washington, USA, in April 2021. Effectiveness of 2 doses of mRNA vaccines against P.1 infection among residents in this outbreak was 75.0% (95% CI 44.5%–88.7%), similar to effectiveness for other pre-Delta variants among long-term care residents.

COVID-19 mRNA vaccines demonstrated high efficacy (>94%) against COVID-19 in clinical trials (*1*,*2*). However, initial observational vaccine effectiveness (VE) estimates against infection among residents of skilled nursing facilities (SNFs), a high-risk population, were lower, 53%–75% (*3*). A local health department in Washington, USA, investigated a COVID-19 outbreak of the P.1 (Gamma) variant in April 2021 in an SNF and estimated VE of 2 mRNA vaccine doses against SARS-CoV-2 infection. The Centers for Disease Control and Prevention reviewed the activity to confirm it was conducted consistent with applicable federal law and organizational policy. This investigation was defined as having met the requirements for public health surveillance as outlined in 45 C.F.R. part 46.102(l) (*2*).

Daily symptom screening of residents and staff had been ongoing in this SNF since March 2020. Routine antigen testing of symptomatic residents with BinaxNOW tests (Abbott Diagnostics, https://www.diagnostics.abbott) was performed upon symptom recognition; routine testing of staff was ongoing. Nucleic acid amplification test (NAAT) confirmation of all positive antigen results and antigen negative results for symptomatic persons was performed. The outbreak index case was a symptomatic fully vaccinated resident identified on April 16, 2021. All residents and staff were tested immediately and again every 3–7 days for the duration of the outbreak period, April 15–May 9, 2021.

We defined a case as a positive SARS-CoV-2 antigen or NAAT result in a resident of the SNF. The local health jurisdiction requested viral whole-genome sequencing (WGS) for all positive specimens. Washington State Department of Health Public Health Laboratories and their partners identified SARS-CoV-2 variant status for individual cases through WGS and recorded cases in the Washington Disease Reporting System.

The SNF conducted vaccination clinics on January 12, February 2, and February 23, 2021. We defined vaccination status as fully vaccinated with 2 doses, if receipt of second vaccine dose was >14 days before the outbreak began (*4*), and unvaccinated if no COVID-19 vaccine had been received before or during the outbreak. We excluded from the VE analysis residents who were partially vaccinated (i.e., who had received 1 vaccine dose or had received a second dose <14 days before the outbreak). We ascertained vaccination status through Washington Immunization Information System and facility medical records. We obtained age, race, ethnicity, and comorbidity information from facility medical records.

We calculated VE for 2 mRNA vaccine doses on the basis of relative risk (RR) of infection in vaccinated versus unvaccinated residents using a log-binomial model and adjusted for potential confounders of age (<85 vs. >85 years) and race (White vs. all other residents with nonmissing race). We used the equation VE = 100% × (1 − RR). We conducted a separate analysis limited to WGS-confirmed P.1 cases to estimate VE against P.1 infection.

Of 63 residents present during the outbreak, 43 (68%) were fully vaccinated with 2 doses and 16 (25%) were unvaccinated; we excluded 4 partially vaccinated residents from the analysis. Thirty-six (84%) of 43 vaccinated residents received vaccination during the onsite clinics. Seven residents (16%) were fully vaccinated at other locations. Nineteen residents tested positive for SARS-CoV-2 during the outbreak (Figure; Appendix Figure); 2 of those were partially vaccinated and excluded from analysis. Of the 17 included outbreak cases, 7 were in fully vaccinated residents. Thirteen (77%) of 17 outbreak cases had WGS data; all were identified as P.1 lineage.

**Figure:**
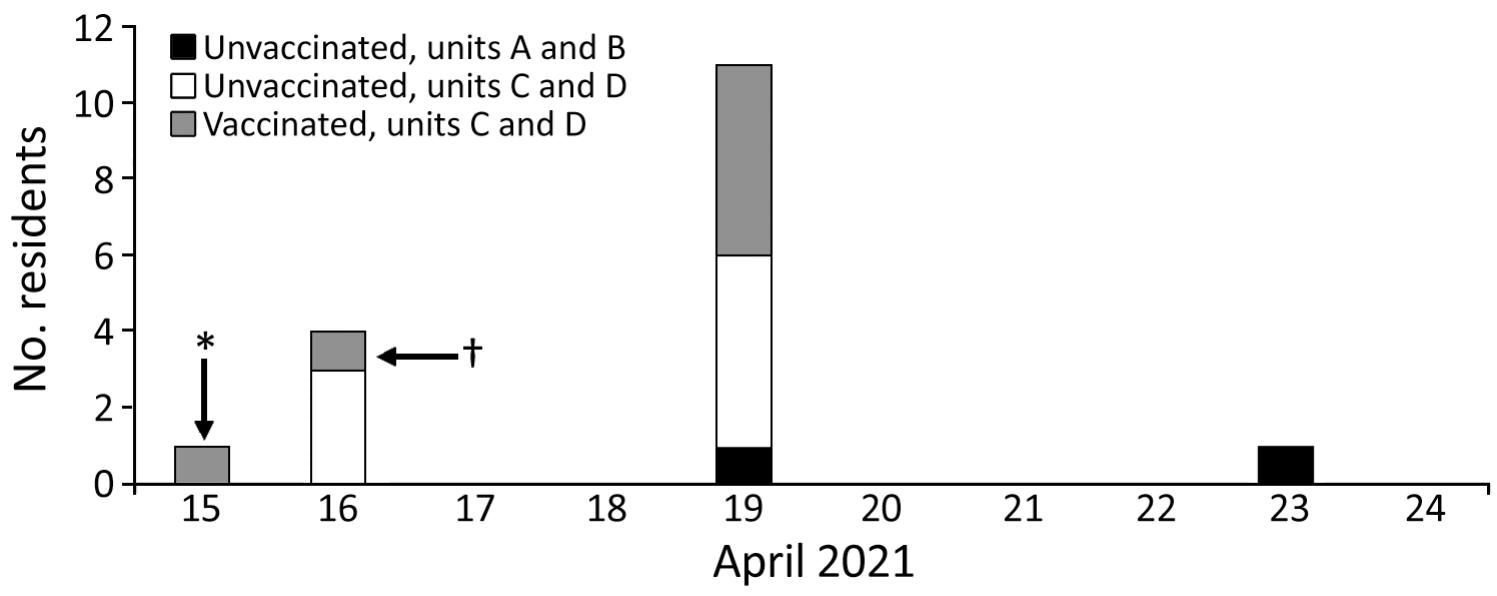
Date of first positive SARS-COV-2 specimen collection among residents in a skilled nursing facility, Washington, April 2021, Cases shown are restricted to the 17 resident cases included in vaccine effectiveness (VE) analysis. Testing was concentrated on point prevalence survey days. Units A and B were long-stay units; units C and D were short-stay units. Asterisk (*) indicates a resident who was discharged from a short-stay unit and later tested positive at an area hospital; dagger (†) indicates a resident who tested positive after symptom screening.

Most of the 59 residents included in the analysis were White (83%) and female (63%); the age range was >60 years (Table). Ethnicity was unknown for 56% of residents. All residents had ≥2 underlying health conditions that may increase risk for severe COVID-19.

**Table:** Characteristics of residents in a skilled nursing facility included in a SARS-CoV-2 vaccine effectiveness analysis, Washington, USA, April 2021*

Characteristic	No. (%) fully vaccinated	No. (%) unvaccinated
Residents present during outbreak	43	16
Sex		
M	15 (35)	7 (44)
F	28 (65)	9 (56)
Age group, y		
60–74	7 (16)	4 (25)
75–84	14 (33)	6 (38)
>85	22 (51)	6 (38)
Race†		
Asian	5 (12)	0
Black or African-American	0	2 (13)
White	36 (84)	13 (81)
Other	1 (2)	0
Unknown	1 (2)	1 (6)
Underlying health conditions		
Hypertension	32 (75)	10 (63)
Neurologic disease	32 (75)	15 (94)
Cardiovascular disease	27 (63)	13 (81)
Diabetes	14 (33)	3 (19)
Asthma, COPD, sleep apnea, other chronic respiratory disease	12 (28)	7 (44)
Obesity	7 (16)	4 (25)
Autoimmune condition	5 (12)	0
Cancer	2 (5)	1 (6)
Immunosuppressive disease or medication	2 (5)	0
End-stage renal disease requiring dialysis	0	1 (6)
Other, nonneurologic condition	42 (98)	16 (100)
>2 underlying conditions	43 (100)	16 (100)
Unit		
Unit A, long-stay unit	15 (35)	4 (25)
Unit B, long-stay unit	16 (37)	4 (25)
Unit C, short-stay unit	10 (23)	4 (25)
Unit D, short-stay unit	2 (5)	4 (25)
History of prior SARS COV-2 infection‡	13 (30)	3 (19)
Tested positive for SARS COV-2 during outbreak period	7 (16)	10 (63)

*Four residents who were partially vaccinated (received 1 dose of COVID-19 vaccine) were excluded from this analysis. COPD, chronic obstructive pulmonary disease. †No residents reported Hispanic or Latino ethnicity. Data for ethnicity was highly missing; 25 (58%) vaccinated residents and 8 (50%) unvaccinated residents were of unknown ethnicity. ‡All previous infections were >3 mo before start of outbreak (before January 13, 2021).

The attack rate in unvaccinated residents was 63% (10/16) versus 16% (7/43) in fully vaccinated residents (adjusted RR 4.0, 95% CI 1.8–8.9). Unadjusted VE against infection was 74.0% (95% CI 43.4%–88.0%). Age-adjusted and race-adjusted VE against infection among 57 residents (excluding 2 residents with unknown race) was 75.0% (95% CI 44.5%–88.7%). Age- and race-adjusted VE against WGS-confirmed P.1 infection among 53 residents (excluding 2 residents with unknown race) was 80.0% (95% CI 46.4%–92.6%). In this outbreak, vaccination was associated with decreased likelihood of infection. Our estimated VE of 75% (95% CI 45%–89%) against infection is consistent with other findings of mRNA VE against infection with other pre-Delta variants among residents of SNFs (*3**–**7*).

The first limitation of our study is that unvaccinated residents might have differed from vaccinated in ways we did not measure, including in the use of mitigation behaviors. In addition, the demographics of residents in this facility may differ from the broader general long-term care resident population.

In conclusion, our evaluation indicates that receiving 2 mRNA vaccine doses was effective in reducing the likelihood of testing positive for SARS-CoV-2 during an outbreak of P.1 lineage variant in an SNF. VE against P.1 is comparable to that against other pre-Delta SARS-CoV-2 variants among long-term care residents.

Appendix Additional information about a study of vaccine effectiveness against SARS-CoV-2 variant P.1 in nursing-facility residents in Washington, USA, April 2021.
