# Supplementary material for: Vaccine Effectiveness against SARS-CoV-2 Variant P.1 in Nursing-Facility Residents, Washington, USA, April 2021
Source: Emerg Infect Dis. 2022 Nov;28(11):2338–41. doi: 10.3201/eid2811.221043 (PMC9622236; doi:10.3201/eid2811.221043)
Supplement: Appendix — Additional information about a study of vaccine effectiveness against SARS-CoV-2 variant P.1 in nursing-facility residents in Washington, USA, April 2021. [file 22-1043-Techapp-s1.pdf]

Article DOI: https://doi.org/10.3201/eid2811.221043

Vaccine Effectiveness Against SARS-CoV-2 Variant P.1 in Nursing-Facility Residents, Washington, USA, April 2021

Appendix

A local health department in Washington State investigated a COVID-19 outbreak of the P.1 (Gamma) variant in April 2021 in a skilled-nursing facility and estimated vaccine effectiveness of 2 mRNA vaccine doses against SARS-CoV-2 infection. Six residents with SARS-CoV-2 infection were hospitalized, 4 of whom were unvaccinated. Onsite vaccination clinics were held on January 12, February 2, and February 23, 2021.

There were 4 deaths among residents with SARS-CoV-2 infection, all of whom were unvaccinated; one decedent was on comfort care at the time of their SARS-CoV-2 diagnosis and declined treatment.

Infection prevention and control measures implemented during the outbreak included increased testing frequency, cohorting of residents by SARS-CoV-2 status, placement of positive and exposed residents on transmission-based precautions, dedicating staff to specific units, and pausing communal dining and activities.

Staff cases were not included in the analysis. Fifty-seven of 68 staff (84%) were fully vaccinated with 2 Pfizer-BioNTech vaccine doses, and 2 staff (3%) were partially vaccinated. Ten of 68 staff (15%) tested positive during the outbreak; none required hospitalization and 6 were fully vaccinated. Whole-genome sequencing was performed for 8; all were identified as P.1 lineage.
